# Three Planctomycetes isolated from biotic surfaces in the Mediterranean Sea and the Pacific Ocean constitute the novel species *Symmachiella dynata* gen. nov., sp. nov. and *Symmachiella macrocystis* sp. nov.

**DOI:** 10.1007/s10482-020-01464-9

**Published:** 2020-08-24

**Authors:** Markus Salbreiter, Muhammad Waqqas, Mareike Jogler, Nicolai Kallscheuer, Sandra Wiegand, Stijn H. Peeters, Anja Heuer, Mike S. M. Jetten, Christian Boedeker, Patrick Rast, Manfred Rohde, Christian Jogler

**Affiliations:** 1 Department of Microbial Interactions, Friedrich-Schiller-University, Jena, Germany; 2 Department of Microbiology, Radboud Universiteit, Nijmegen, The Netherlands; 3 Institute for Biological Interfaces 5, Karlsruhe Institute of Technology, Eggenstein- Leopoldshafen, Germany; 4 Leibniz Institute DSMZ, Braunschweig, Germany; 5 Central Facility for Microscopy, Helmholtz Centre for Infection Research, Braunschweig, Germany

**Keywords:** *Planctomycetes*, Marine bacteria, Mallorca, California, Panarea, Aggregation, Crateriform structures, Budding, *Planctomycetaceae*

## Abstract

*Planctomycetes* is a phylum of environmentally important bacteria, which also receive significant attention due to their fascinating cell biology. Access to axenic Planctomycete cultures is crucial to study cell biological features within this phylum in further detail. In this study, we characterise three novel strains, Mal52^T^, Pan258 and CA54^T^, which were isolated close to the coasts of the islands Mallorca (Spain) and Panarea (Italy), and from Monterey Bay, CA, USA. The three isolates show optimal growth at temperatures between 22 and 24 °C and at pH 7.5, divide by polar budding, lack pigmentation and form strong aggregates in liquid culture. Analysis of five phylogenetic markers suggests that the strains constitute two novel species within a novel genus in the family *Planctomycetaceae*. The strains Mal52^T^ (DSM 101177^T^ = VKM B-3432^T^) and Pan258 were assigned to the species *Symmachiella dynata* gen nov., sp. nov., while strain CA54^T^ (DSM 104301^T^ = VKM B-3450^T^) forms a separate species of the same genus, for which we propose the name *Symmachiella macrocystis* sp. nov.

## Introduction

Planctomycetes were first discovered in 1924 and mistakenly acknowledged as eukaryotes (Gimesi 1924), but later reclassified as bacteria (Hirsch 1972). Planctomycetes are ubiquitous bacteria dwelling in marine, limnic and soil environments, in which they play an important role in the global carbon and nitrogen cycle (Wiegand et al. 2018). The eponymous phylum *Planctomycetes* is part of the PVC superphylum, which additionally includes the phyla *Verrucomicrobia, Chlamydiae* and other sister phyla. The PVC superphylum has medical and biotechnological relevance (Rivas-Marin and Devos 2018; Wagner and Horn 2006). According to the current taxonomy, the phylum *Planctomycetes* is divided into the classes *Phycisphaerae* and *Planctomycetia*. *Candidatus* Brocadiae might very well form a third class within the phylum, but no axenic cultures have been obtained from this class so far (Kartal et al. 2013). Known members of the class *Planctomycetia* divide by budding, while binary fission was observed as cell division mode in the class *Phycisphaerae*. The class *Planctomycetia* was recently re-organised and is now further subdivided into the orders *Isosphaerales*, *Gemmatales*, *Planctomycetales* and *Pirellulales* (Dedysh et al. 2020b).

Planctomycetes can be found in various habitats on earth and can even be amongst the most abundant phyla in bacterial communities on biotic surfaces, e.g. on marine macroscopic phototrophs (Bengtsson and Øvreås 2010; Bondoso et al. 2014,  2015, 2017; Lage and Bondoso 2014). Given the oligotrophic nature of seawater, Planctomycetes are suggested to use complex substrates secreted by phototrophs as sources of carbon and energy (Jeske et al. 2013; Lachnit et al. 2013). Indeed, in silico genome analyses point towards higher numbers of carbohydrate-active enzymes encoded by Planctomycetes (Ivanova et al. 2017; Wallner et al. 2005; Wegner et al. 2013). In this context, pili originating from crateriform structures and an enlarged periplasmic space are discussed to be part of a specific uptake system, which may allow intracellular digestion of entire high-molecular weight sugar molecules (Boedeker et al. 2017). If true, this strategy is a decisive advantage over the use of extracellular enzymes for degradation since the latter strategy would provide easily degradable carbon sources to competitors.

Despite the assumed presence of such catabolic systems, the high abundance of Planctomycetes is still unexpected given their slow growth compared to many other heterotrophic bacteria competing with Planctomycetes for ‘nutrient-rich’ ecological niches (Frank et al. 2014; Wiegand et al. 2018). The potential for production of small molecules with antimicrobial properties may also play a decisive role in such environments (Graça et al. 2016; Jeske et al. 2013).

Morphologically, Planctomycetes have been suggested to possess uncommon traits compared to canonical bacteria. Different traits, including the lack of peptidoglycan (König et al. 1984), a compartmentalised cell plan (Lindsay et al. 1997), a nucleus-like structure (Fuerst and Webb 1991) and endocytosis-like uptake (Lonhienne et al. 2010) have been proposed. Some of these traits were found not to be entirely accurate. The compartmentalised cell plan turned out to be invaginations of the cytoplasmic membrane (Acehan et al. 2013; Boedeker et al. 2017), while presence of peptidoglycan was demonstrated (Jeske et al. 2015; Van Teeseling et al. 2015). The cell plan of Planctomycetes was revised based on the use of novel microscopy techniques and genetic tools, and the cell envelope architecture is now considered similar to that of Gram-negative bacteria (Devos 2014; Jogler et al. 2011; Jogler and Jogler 2013; Rivas-Marin et al. 2016). However, Planctomycetes are still unusual. They e.g. lack canonical divisome proteins including the otherwise essential FtsZ (Jogler et al. 2012; Pilhofer et al. 2008) and 40–55% of the proteins encoded in planctomycetal genomes are of unknown function.

For extending the current collection of Planctomycetes available as axenic cultures, here we describe three novel closely related strains, which we isolated from algae close to the island Mallorca, from seagrass leaves close to the island Panarea and from the kelp forest at Monterey Bay in California, USA.

## Materials and methods

### Isolation of the novel strains

The three novel strains Mal52^T^, Pan258 and CA54^T^ were isolated as previously described (Wiegand et al. 2020). Strain CA54^T^ was isolated from a *Macrocystis pyrifera* kelp forest at Monterey Bay, CA, USA on November 28th, 2014 (exact location: 36.619 N 121.901 W). Strain Mal52^T^ was obtained from algae in the Mediterranean Sea close to S’Arenal, Mallorca, Spain (exact location: 39.5126 N 2.7470 E) on September 23rd, 2014. Strain Pan258 was isolated from seagrass leaves growing next to a natural gas escape of the hydrothermal vent system close to Panarea Island (exact location: 38.6457 N 15.0772 E), which were sampled on September 9th, 2013. In order to prevent fungal growth, pieces of kelp, alga and seagrass were initially rinsed with 100 mg/L cycloheximide dissolved in sterile-filtered natural seawater and subsequently swabbed over plates with solidified M1H NAG ASW medium (Kallscheuer et al. 2019a) containing 8 g/L gellan gum, 1000 mg/L streptomycin, 200 mg/L ampicillin and 20 mg/L cycloheximide. The plates were incubated at 20 °C for at least six weeks. Colonies obtained were restreaked on fresh plates, which were used to inoculate liquid M1H NAG ASW medium. Sequencing of the 16S rRNA gene of the colonies was performed according to a previously published protocol to ensure that novel strains are members of the phylum *Planctomycetes* (Rast et al. 2017).

### Light and electron microscopy

Phase contrast and scanning electron microscopic analyses were performed as described in a previous study (Boersma et al. 2019).

### Genome information and genome-based analysis of the carbon metabolism

The genome sequences of the three novel isolates are available from GenBank under accession numbers CP036270 (Pan258), CP036276 (Mal52^T^) and SJPP00000000 (CA54^T^). The 16S rRNA gene sequences can be found under accession numbers MK554517 (Pan258), MK554513 (Mal52^T^) and MK554522 (CA54^T^). DNA isolation and genome sequencing are part of a previous study (Wiegand et al. 2020). The genome-based analysis of the carbon metabolism of the novel isolates was performed as previously described (Rivas-Marin et al. 2020).

### Physiological analyses

The pH optimum for growth was determined in M1H NAG ASW medium with 100 mM of the following buffers: 2-(*N*-morpholino)ethanesulfonic acid (MES) for pH 5.0 and 6.0, 4-(2-hydroxyethyl)-1-piperazineethanesulfonic acid (HEPES) for pH 7.0, 7.5 and 8.0 and *N*-cyclohexyl-2-aminoethanesulfonic acid (CHES) for pH 9.0 and 10.0. The cultures were incubated at 28 °C. The temperature optimum for growth was determined by cultivation at temperatures ranging from 10 to 40 °C at pH 8.0. All cultivations were performed in triplicates and growth was assessed by measuring the optical density at 600 nm (OD_600_). Growth rates for each tested condition were calculated by plotting ln(OD_600_), the natural logarithm of average OD_600_ values from biological triplicates, against the cultivation time. The slope of the linear range of this plot (at least five data points) was used as maximal growth rate µ_max_ (in h^− 1^). Generation times t_d_ (in h) were calculated using the formula t_d_ = ln(2)/µ_max_.

### Phylogenetic analyses

Maximum likelihood 16S rRNA gene sequence-based phylogeny was computed for the novel strains, the described type strains of all planctomycetal species (as of June 2020), including recently published strains (Boersma et al. 2019; Dedysh et al. 2020a, b; Kallscheuer et al. 2019a, b, c, 2020a, b; Kohn et al. 2020; Peeters et al. 2020; Wiegand et al. 2020). The alignment of 16S rRNA gene sequences was performed with SINA (Pruesse et al. 2012). A maximum likelihood approach with 1000 bootstraps, nucleotide substitution model GTR, gamma distribution and estimation of proportion of invariable sites (Stamatakis 2014) was used. The outgroup consisted of three 16S rRNA gene from strains outside of the phylum *Planctomycetes*, but still part of the PVC superphylum. For the multi-locus sequence analysis (MLSA), the unique single-copy core genome of the analysed genomes was determined with proteinortho5 (Lechner et al. 2011) with the ‘selfblast’ option enabled. The protein sequences of the resulting orthologous groups were aligned using MUSCLE v.3.8.31 (Edgar 2004). After clipping, partially aligned *C*- and *N*-terminal regions and poorly aligned internal regions were filtered using Gblocks (Castresana 2000). The final alignment was concatenated and clustered using the maximum likelihood method implemented by RAxML (Stamatakis 2014) with the ‘rapid bootstrap’ method and 500 bootstrap replicates. Five planctomycetal genomes from the order *Pirellulales* served as outgroup. The *rpoB* gene sequences were taken from publicly available online databases and sequence identities were determined as previously described (Bondoso et al. 2013). The average nucleotide identity (ANI) was calculated with OrthoANI (Lee et al. 2016). The average amino acid identity (AAI) was calculated using the aai.rb script of the enveomics collection (Rodriguez-R and Konstantinidis 2016) and percentage of conserved proteins (POCP) was calculated as described (Qin et al. 2014).

## Results and discussion

### Phylogenetic inference

**Fig. 1:**
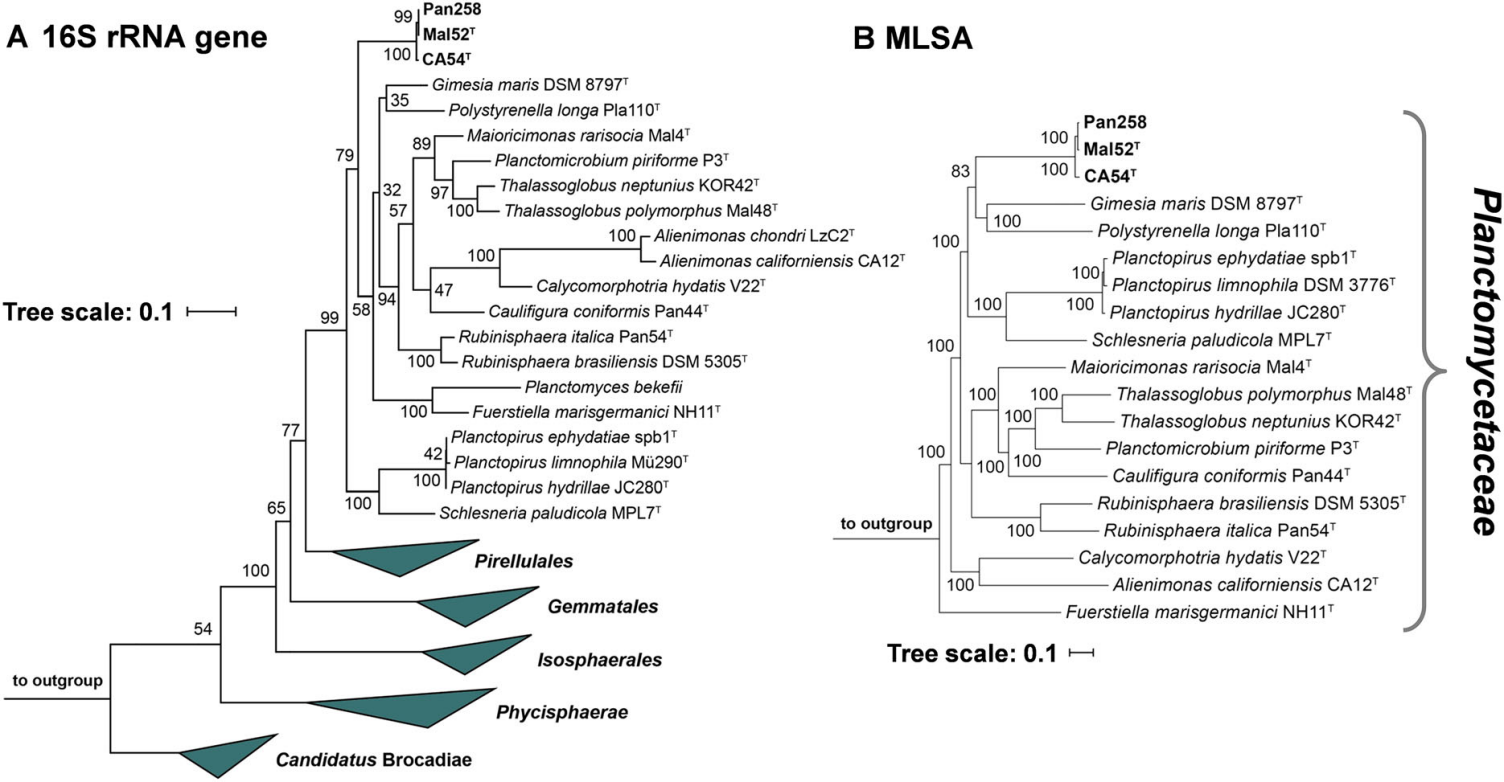
Maximum likelihood 16S rRNA gene sequence-(**a**) and MLSA-based phylogenetic trees (**b**) depicting the positions of strains Mal52^T^, Pan258 and CA54^T^. Phylogeny was calculated as described in the Material and methods section. Bootstrap values after 1000 re-samplings (500 re-sampling for MLSA analysis) are given at the nodes (in %). The tree scale (branch length values) represents the mean expected rates of substitution per site. The outgroups consist of three 16S rRNA genes from the PVC superphylum outside of the phylum *Planctomycetes* (**a**) and of members of the order *Pirellulales* (**b**)

**Fig. 2:**
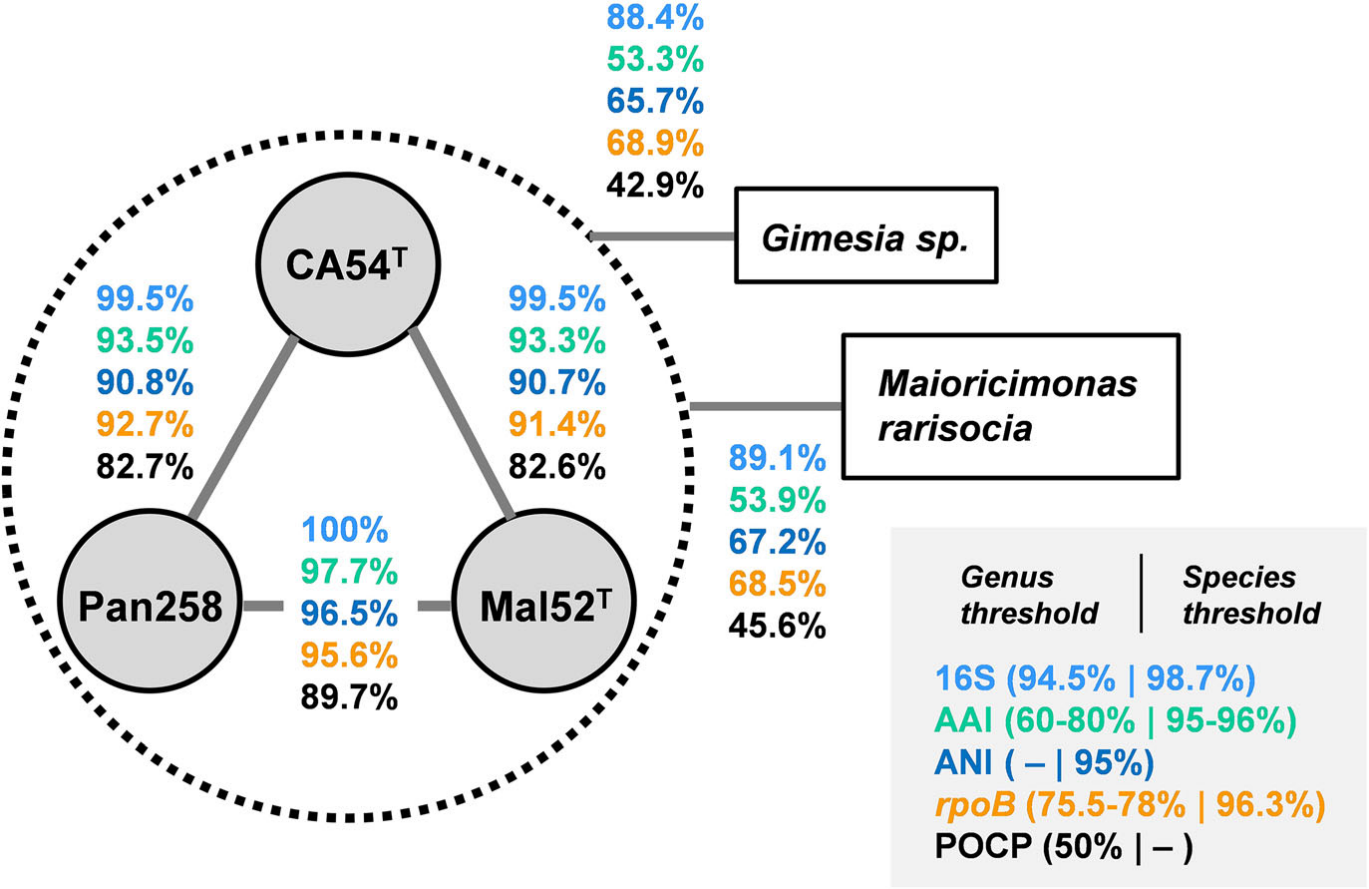
Comparison of phylogenetic markers for delineation of the novel isolates Mal52^T^, Pan258 and CA54^T^. Methods used: 16S rRNA gene identity (16S), average amino acid identity (AAI), average nucleotide identity (ANI), *rpoB* gene identity and percentage of conserved proteins (POCP)

In the phylogenetic trees obtained after analysis of 16S rRNA genes and MLSA, the strains Mal52^T^, Pan258 and CA54^T^ form a monophyletic cluster within the family *Planctomycetaceae* (Fig. 1). Both trees as well as five analysed phylogenetic markers suggest *Maioricimonas rarisocia* Mal4^T^ (Rivas-Marin et al. 2020) and *Gimesia maris* (Scheuner et al. 2014) as current closest relatives of the three novel isolates. Based on this finding, we analysed 16S rRNA gene sequence similarity, *rpoB* gene similarity, AAI and POCP to check whether the novel isolates belong to one of the two genera. The three strains share a minimal 16S rRNA gene sequence identity of 89.1% with *M. rarisocia* Mal4^T^ and 88.4% with *Gimesia* sp. Both values are significantly below the proposed genus threshold of 94.5% (Yarza et al. 2014), indicating that these strains belong to a separate, yet undescribed genus in the family *Planctomycetaceae* (Fig. 2). This finding is also supported by analyses of *rpoB* similarity, AAI and POCP, since comparison of the three novel isolates with members of the above-mentioned genera yielded values below the respective genus thresholds of 75.5–78% for *rpoB* (Kallscheuer et al. 2019c), 60% for AAI (Konstantinidis and Tiedje 2005) and approximately 50% for POCP (Qin et al. 2014) (Fig. 2). ANI values in a range of 65–67% and thus far below the species threshold of 95% (Kim et al. 2014) thereby also ensure that the novel strains do not belong to any described species.

Next, we compared the strains Mal52^T^, Pan258 and CA54^T^ against each other to check if they belong to separate species. It turned out that strains Mal52^T^ and Pan258 have a 100% identical 16S rRNA gene sequence, indicating that they belong to the same species. This assumption is supported by an ANI of 96.5% above the species threshold of 95% and an AAI of 97.7% (proposed species threshold of 95–96%) (Konstantinidis and Tiedje 2005). Only the *rpoB* similarity of 95.6% is below, but still close to the species threshold of 96.3% (Bondoso et al. 2013) (Fig. 2). In particular due to an identical 16S rRNA gene sequence, we conclude that the strains Mal52^T^ and Pan258 belong to the same species. In constrast, comparison of either of these two strains with strain CA54^T^ yielded identity values for AAI and ANI significantly below the species threshold values (Fig. 2). Although strain CA54^T^ shares an identity of 99.5% on 16S rRNA gene sequence level (species threshold 98.7%), we decided to assign it to a separate species. This decision is based on previous observations that this threshold is not always applicable for members of the class *Planctomycetia* and that strains can belong to separate species despite 16S rRNA gene sequence similarities above the threshold (Kohn et al. 2020). Taken together, the phylogenetic analysis suggests that the three strains represent two novel species of a novel genus within the family *Planctomycetaceae*.

### Morphological and physiological analyses

**Fig. 3:**
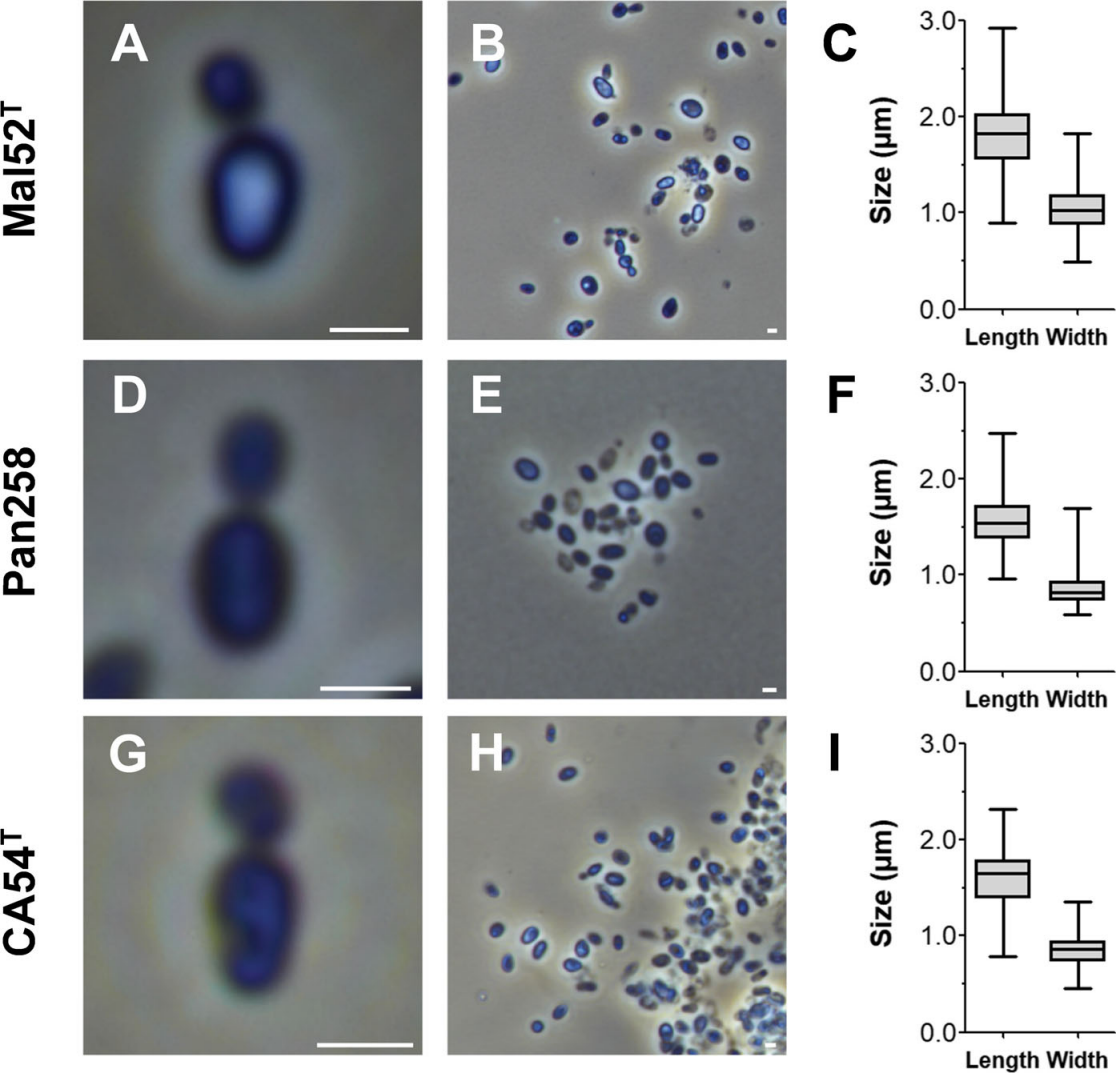
Light microscopic photographs and cell size plots of the novel strains. The cell sizes were determined by manually measuring at least 100 cells or by applying a semi-automated object count tool. The scale bar is 1 µm

**Fig. 4:**
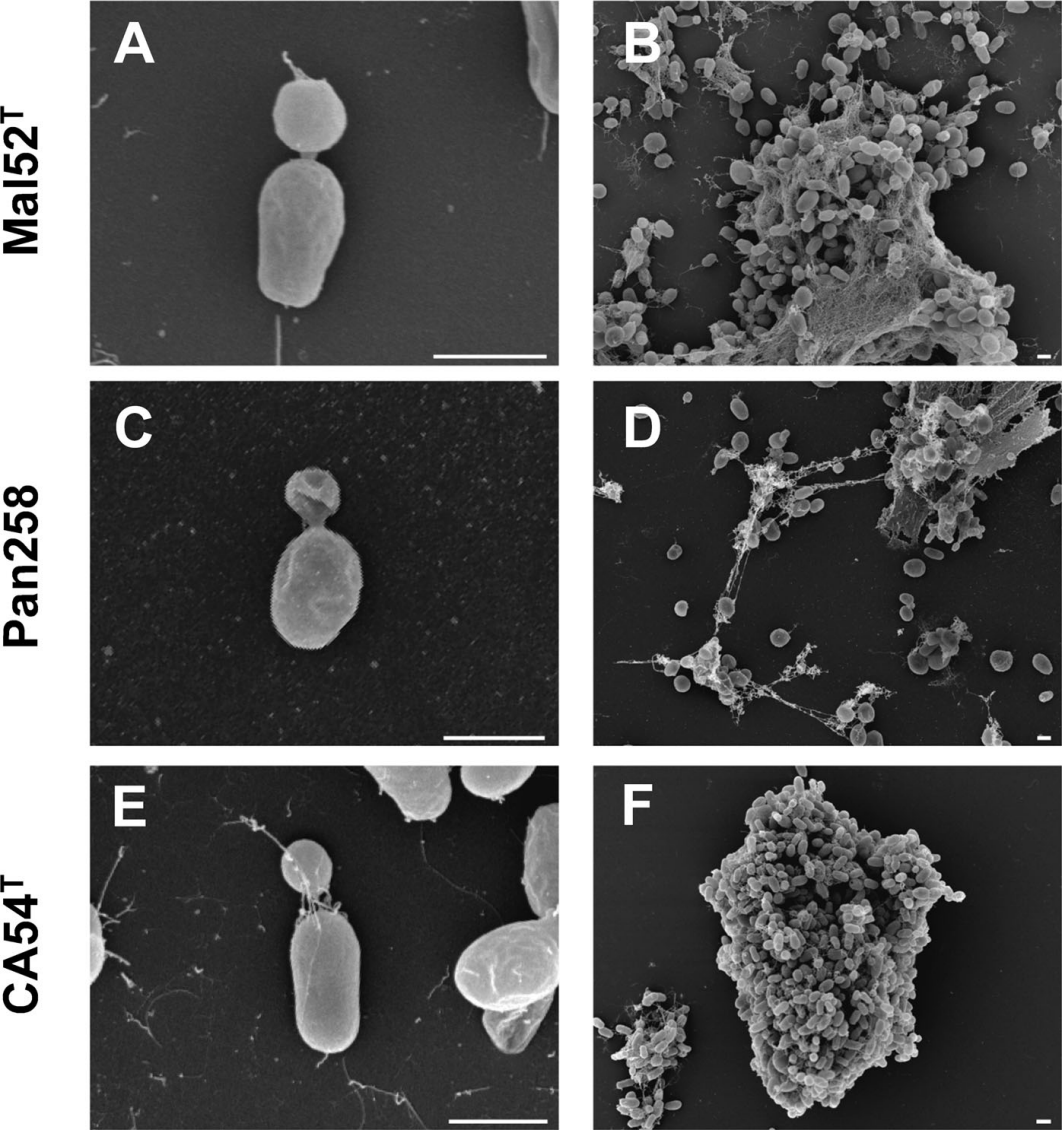
Scanning electron micrographs of the novel isolates. The scale bar indicates 1 µm

For microscopic analyses of the three isolated strains, cells were harvested during the exponential growth phase. Detailed information on morphology, cell division and motility is summarised in Table 1. The current closest relatives *M. rarisocia* and *G. maris* were chosen for comparison. Strain Mal52^T^ (Figs. 3a–c, 4a,b) and strain Pan258 (Figs. 3d–f, 4c,d) form white colonies on plates and cells have an ovoid to pear-shaped morphology. Strain CA54^T^ displayed white- to cream-coloured colonies. Cells of this strain were ovoid to pear-shaped, but also rod-shaped cells were observed (Figs. 3g–i,  4e,f); a phenotype that was not found for the other two isolates. The cell shape of the novel isolates differs from spherical *G. maris* cells. While the average cell size of strains Mal52^T^, Pan258 and CA54^T^ turned out to be similar (1.6–1.8 × 0.8–1.0 µm) (Fig. 3c,f,i), all three are slightly smaller than cells of *M. rarisocia* Mal4^T^.

**Table 1:** Phenotypic and genotypic features of strains Mal52^T^, Pan258 and CA54^T^ in comparison to their current closest relatives

Characteristics	Mal52^T^	Pan258	CA54^T^	*Mairicimonas* *rarisocia* Mal4^T^	*Gimesia maris* DSM 8797^T^
*Phenotypic features*					
Colour	White	White	White to cream	Orange	White
Size	1.8 × 1.0 µm	1.6 × 0.9 µm	1.6 × 0.8 µm	2.0 × 1.4 µm	0.4–1.5 µm
Shape	Ovoid to pear-shaped	Ovoid to pear-shaped	Ovoid, pear-shaped or rod-shaped	Pear-shaped	Spherical to ovoid
Aggregates	Yes, big	Yes	Yes	Rarely	Yes
Temperature range (optimum) (°C)	15–30 (24)	15–30 (24)	15–24 (22)	10–39 (31)	6–37 (30–33)
pH range (optimum)	5.5–9.5 (7.5)	5.5–9.5 (7.5)	6.0–9.0 (7.5)	6.5–9.0 (7.5)	n.d.
Division	Budding	Budding	Budding	Budding	Budding
Dimorphic lifestyle	n.o.	n.o.	n.o.	n.o.	Yes
Motility	Yes	Yes	Yes	n.o.	Yes
Crateriform structures	Yes	n.o.	n.o.	Yes, overall	n.o.
Fimbriae	Matrix or fibre	Matrix or fibre	Polar matrix or fibre	Matrix or fibre	Matrix or fibre
Capsule	n.o.	n.o.	n.o.	n.o.	n.o.
Stalk	n.o.	n.o.	n.o.	Yes	Yes
Holdfast structure	n.o.	n.o.	n.o.	n.o.	Yes
*Genomic features*					
Genome size (bp)	7,767,809	7,597,262	7,574,949	7,744,989	7,816,689
Plasmids (bp)	no	n.o.	no	no	no
G + C (%)	55.3	55.3	55.2	63.4	50.4
Completeness (%)	96.55	96.55	96.55	98.28	98.28
Contamination (%)	3.45	3.45	3.45	0	1.72
Protein-coding genes	6143	6011	6082	5829	5986
Hypothetical proteins	2510	2486	2509	2257	2400
Protein-coding genes/Mb	791	791	803	753	766
Coding density (%)	85.6	85.4	86.1	85.9	86.9
16S rRNA genes	2	1	1	2	2
tRNA genes	71	72	78	55	66

n.o. not observed

The lack of pigmentation indicates the incapability of the strains to form carotenoids. In that regard, they are similar to *G. maris*, but differ from the orange pigmentation of *M. rarisocia*. A strong tendency to aggregate and biofilm formation was observed. This is a considerable difference to *M. rarisocia* Mal4^T^, which mostly occurs in the form of single cells and only in rare cases forms aggregates. Crateriform structures could only be observed on the surface of Mal52^T^ cells, however we cannot exclude the presence in case of the other two strains. Cells of all three strains are motile and divide by polar budding.

During cultivation experiments, strains Mal52^T^ and Pan258 grew over a temperature range of 15–30 °C and a pH range of 5.5–9.5 (Table 1). Strain CA54^T^ showed a similar pH range and all three strains showed optimal growth at pH 7.5. The optimum temperature for growth falls between 22 and 24 °C and is thus considerably lower than observed for *M. rarisocia* and *G. maris* (30-33 °C). The novel isolates are slow-growing strains with maximal growth rates between 0.005 and 0.01 h^− 1^ (generation times of 70–140 h) in M1H NAG ASW medium.

### Genomic characteristics

Genome characteristics are listed in Table 1. The three novel isolates and the two species chosen for comparison have very similar genome sizes of 7.6–7.8 Mb. Not surprisingly, numbers of protein-coding genes (5,829-6,143), protein-coding genes per Mb (753–803) and coding densities (85.4–86.9%) are similar. In contrast, the novel strains can be clearly differentiated from *M. rarisocia* and *G. maris* by differences in the DNA G + C content of their genomes (Table 1). Strain Mal52^T^ has two copies of the 16S rRNA gene, while only a single 16S rRNA gene was found in the genomes of the other two novel isolates. None of the compared strains harbors plasmids. In all five genomes 39–41% of the automatically annotated genes code for proteins with unknown function. These values are in the lower range of 40–55% observed in genomes of Planctomycetes sequenced so far.

### Genome-based analysis of enzymes participating in the central carbon metabolism

Based on the genomes of strains Pan258, Mal52^T^ and CA54^T^, the presence of key metabolic enzymes of the central carbon metabolism was analysed. The analysis included glycolytic pathways (Embden–Meyerhof–Parnas pathway or Entner–Doudoroff pathway), the tricarboxylic acid (TCA) cycle, gluconeogenesis and anaplerotic reactions (Table 2). All three strains contain genes coding for enzymes involved in glycolysis, both for the Embden–Meyerhof–Parnas pathway and the Entner–Doudoroff pathway. In addition, key enzymes for sugar degradation via the pentose phosphate pathway were found in all three strains. This was not surprising since important precursors for amino acid and nucleotide biosynthesis branch off from the pentose phosphate pathway and auxotrophies occur in case that this pathway is non-functional. Further analysis showed that genes coding for all enzymes of the TCA cycle could be found in each strain. Genes coding for enzymes required for conversion of oxaloacetate to phosphoenolpyruvate and for C1-dephosphorylation of fructose-1,6-bisphosphate as key steps of a functional gluconeogenesis were identified. Thus, all three strains should be capable of de novo sugar biosynthesis. In contrast, the glyoxylate shunt, an important anaplerotic pathway during growth on acetate or fatty acids, is absent in all three strains, which appears to be a common feature of Planctomycetes.

**Table 2:** Genome-based analysis of the central carbon metabolism of Pan258, Mal52^T^ and CA54^T^

Enzyme	EC number	Gene	Pan258	Mal52^T^	CA54^T^
*Glycolysis*					
Glucose-6-phosphate isomerase	5.3.1.9	*pgi*	Pan258_13410	Mal52_12810	CA54_06070
ATP-dependent 6-phosphofructokinase isoenzyme 1	2.7.1.11	*pfkA*	Pan258_34720	Mal52_35820	CA54_51790
Fructose-bisphosphate aldolase class 2	4.1.2.13	*fbaA*	Pan258_52530	Mal52_53960	CA54_27120
Triosephosphate isomerase	5.3.1.1	*tpiA*	Pan258_47170	Mal52_48110	CA54_32530
Glyceraldehyde-3-phosphate dehydrogenase	1.2.1.12	*gapA*	Pan258_58190	Mal52_59510	CA54_21220
Phosphoglycerate kinase	2.7.2.3	*pgk*	Pan258_26210	Mal52_26450	CA54_43030
2,3-Bisphosphoglycerate-independent phosphoglycerate mutase	5.4.2.12	*gpmI*	Pan258_57260	Mal52_58640	CA54_22530
2,3-Bisphosphoglycerate-dependent phosphoglycerate mutase	5.4.2.11	*gpmA*	Pan258_43930	Mal52_45580	CA54_34890
Enolase	4.2.1.11	*eno*	Pan258_16870	Mal52_17050	CA54_02580
			Pan258_24910	Mal52_25160	CA54_44300
Pyruvate kinase I	2.7.1.40	*pykF*	Pan258_33900	Mal52_34970	CA54_50970
Pyruvate dehydrogenase E1 component	1.2.4.1	*aceE*	Pan258_32650	Mal52_33710	CA54_49670
Dihydrolipoyllysine-residue acetyltransferase component of pyruvate dehydrogenase complex	2.3.1.12	*aceF*	Pan258_32660	Mal52_33720	CA54_49680
*Gluconeogenesis*					
Phosphoenolpyruvate synthase	2.7.9.2	*ppsA*	N	N	N
Pyruvate, phosphate dikinase	2.7.9.1	*ppdK*	Pan258_40190	Mal52_41760	CA54_57640
Pyruvate carboxylase	6.4.1.1	*pyc*	Pan258_31100	Mal52_32150	CA54_58530
Phosphoenolpyruvate carboxykinase (ATP)	4.1.1.49	*pckA*	Pan258_48000	Mal52_48950	CA54_31700
Phosphoenolpyruvate carboxykinase (GTP)	4.1.1.32	*pckG*	N	N	N
Phosphoenolpyruvate carboxykinase (diphosphate)	4.1.1.38	*PEPCK*	Pan258_57260	Mal52_58640	CA54_22530
Fructose-1,6-bisphosphatase class 2	3.1.3.11	*glpX*	N	N	N
Fructose-1,6-bisphosphatase class 1	3.1.3.11	*fbp*	Pan258_26740	Mal52_26990	CA54_42510
Pyrophosphate:fructose 6-phosphate 1-phosphotransferase	2.7.1.90	*pfp*	Pan258_19750	Mal52_20120	CA54_49300
*Pentose phosphate pathway*					
Glucose-6-phosphate 1-dehydrogenase	1.1.1.49	*zwf*	Pan258_13960	Mal52_14020	CA54_05160
6-Phosphogluconolactonase	3.1.1.31	*pgl*	Pan258_20660	Mal52_21030	CA54_48440
6-Phosphogluconate dehydrogenase, decarboxylating	1.1.1.44	*gndA*	Pan258_19770	Mal52_20140	CA54_49280
Transketolase 2	2.2.1.1	*tktB*	Pan258_36350, Pan258_36360	Mal52_37790, Mal52_37800	CA54_53330, CA54_53340
Transaldolase B	2.2.1.2	*talB*	Pan258_08210	Mal52_07650	CA54_11050
*Entner-Doudoroff pathway*					
KHG/KDPG aldolase	4.1.2.14	*eda*	Pan258_14040	Mal52_14100	CA54_05100
Phosphogluconate dehydratase	4.2.1.12	*edd*	Pan258_40810	Mal52_42460	CA54_38120
*TCA cycle*					
Citrate synthase	2.3.3.16	*gltA*	Pan258_00560	Mal52_00570	CA54_18160
Aconitate hydratase A	4.2.1.3	*acnA*	Pan258_21500	Mal52_21950	CA54_47570
Isocitrate dehydrogenase [NADP]	1.1.1.42	*icd*	Pan258_54730	Mal52_56040	CA54_25060
2-oxoglutarate dehydrogenase E1 component	1.2.4.2	*sucA*	Pan258_54450	Mal52_55760	CA54_25310
Dihydrolipoyllysine-residue succinyltransferase component of 2-oxoglutarate dehydrogenase complex	2.3.1.61	*sucB*	Pan258_17100	Mal52_17290	CA54_02340
Succinate—CoA ligase [ADP-forming] subunit alpha	6.2.1.5	*sucD*	Pan258_35790	Mal52_36710	CA54_52680
Succinate—CoA ligase [ADP-forming] subunit beta	6.2.1.5	*sucC*	Pan258_35780	Mal52_36700	CA54_52670
Succinate dehydrogenase flavoprotein subunit	1.3.5.1	*sdhA*	Pan258_24540	Mal52_24830	CA54_44660
Succinate dehydrogenase iron-sulfur subunit	1.3.5.1	*sdhB*	Pan258_24530	Mal52_24820	CA54_44670
Succinate dehydrogenase cytochrome b556 subunit	1.3.5.1	*sdhC*	Pan258_24550	Mal52_24840	CA54_44650
Fumarate hydratase class I, an/aerobic	4.2.1.2	*fumA/B*	N	N	N
Fumarate hydratase class II	4.2.1.2	*fumC*	Pan258_33220	Mal52_34290	CA54_50280
Malate dehydrogenase	1.1.1.37	*mdh*	Pan258_14990	Mal52_15170	CA54_04360
*Glyoxylate shunt*					
Isocitrate lyase	4.1.3.1	aceA	N	N	N
Malate synthase G	2.3.3.9	glcB	N	N	N

The analysis is based on the genome accession numbers given in the Material and methods section. Enzymes not identified during the genome-based analysis are indicated by an ‘N’

Taken together, phylogenetic inference as well as morphological, physiological and genomic analyses suggest that the three novel isolates represent two novel species of a novel genus in the family *Planctomycetaceae*. We thus propose to introduce the genus *Symmachiella* gen. nov. Strains Mal52^T^ and Pan258 are assigned to the species *Symmachiella dynata* sp. nov. and CA54^T^ to *Symmachiella macrocystis* sp. nov. Strains Mal52^T^ and CA54^T^ represent the respective type strains of the novel species.

### ***Symmachiella*gen. nov.**

*Symmachiella* (Sym.ma.chi.el’la N.L. fem. n. *Symmachiella* dim. of Gr. *symmachia* a union, an alliance; bacteria that aggregate). Members of the genus have a cell envelope architecture resembling that of Gram-negative bacteria, are aerobic, neutrophilic, mesophilic and heterotrophic. Cells divide by polar budding and form strong aggregates. Species of the genus lack pigmentation. The DNA G + C content is around 55%. The genus is part of the family *Planctomycetaceae*, order *Planctomycetales*, class *Planctomycetia*, phylum *Planctomycetes*. The type species of the genus is *Symmachiella dynata*.

### ***Symmachiella dynata*sp. nov.**

*Symmachiella dynata* (dy.na’ta. N.L. fem. adj. dynata of Gr. dynate strong, intense; corresponding to the strong cohesion between the cells). In addition to the genus characteristics, cells of the species are ovoid or pear-shaped. Cells of the type strain grow between 10 and 30 °C (optimum 24 °C) and at pH 5.0 to 9.5 (optimum pH 7.5). The DNA G + C content of the type strain is 55.3%. The type strain is Mal52^T^ (DSM 101177^T^ = VKM B-3432^T^), which was isolated from an alga close to the coast of S’Arenal on the island Mallorca, Spain. Strain Pan258 (DSM 103143 = VKM B-3436) is an additional member of the novel species.

### ***Symmachiella macrocystis*sp. nov.**

*Symmachiella macrocystis* (ma.cro.cys’tis. N.L. gen. n. macrocystis of *Macrocystis*; corresponding to the isolation of the strain from the giant kelp *Macrocystis pyrifera*). In addition to the genus characteristics, the cell shape is not uniform and can range from ovoid to rod-shape. The type strain is CA54^T^ (DSM 104301^T^ = VKM B-3450^T^), isolated from the giant bladder kelp *Macrocystis pyrifera* in Monterey Bay, California, USA. Growth of the type strain was observed at a temperature range of 15–24 °C (optimum at 22 °C) and at pH 6.5–9.5 (optimum at pH 7.5). The DNA G + C content of the type strain is 55.2%.
